# Practical Chemistry and Pharmacy

**Published:** 1894-03

**Authors:** 

**Affiliations:** LaGrange, Ga.


### *SELECTIONS AND ABSTRACTS.*

### PRACTICAL CHEMISTRY AND PHARMACY.

Edited by HENRY R. SLACK, M. D., Ph. M., LaGrange, Ga.

### A Physician's Opinion on the Changes in Strength of the Preparations of the New United States Pharmacopœia.

Acetum opii is about one-tenth weaker than formerly.

Phosphoric acid is nearly twice as strong as formerly. It is, however, to be noted that this change in phosphoric acid does not affect the physician in his use of the dilute acid, as this is prepared by a new proportional formula, so as to keep its strength the same as before.

Sulphuric acid is apparently a little weaker than formerly, but not really so, as it has not in the past actually attained the standard fixed for it.

Sulphurous acid is nearly twice as strong as formerly.

Calx chlorata is two-fifths stronger than formerly.

Calx sulphurata is nearly twice as strong as formerly.

All decoctions and infusions not specifically mentioned in the Pharmacopœia are only one-half as strong as formerly. (By an error in writing the tables on page 58 of the Pharmacopœia these are made to appear as being four times as strong as they are in reality.)

Liquor sodæ chloratæ is about one-fourth stronger than formerly.

Pepsin is required to have a digestive power over albumen of not less than 1 in 3,000.

Saccharated pepsin is six times as strong as formerly, and one-tenth as strong as pepsin.

Tincture of cannabis indica is one-fifth weaker than formerly.

Tincture of colchicum seed, digitalis, henbane, lobelia and belladonna have been so slightly altered in strength as to make no appreciable difference in their exhibition.

Tincture of cubeb is only half as strong as formerly.

Tincture of gelsemium is one-seventh stronger than formerly.

Tincture of musk is only one-half as strong as formerly.

Tincture of nux vomica, previously without any effective standard, must now contain three-tenths of one per cent. of total alkaloids, while the extract of the same drug must contain fifteen per cent. of total alkaloids, and its fluid extract must contain one and a half grains total alkaloids in 100 c. c.

Tincture of opium, tincture of deodorized opium and camphorated tincture of opium have suffered inappreciable increase of strength.

Tincture of physostigma is about twice as strong as formerly.

Tincture of stramonium seed is nearly twice as strong as formerly.

Tincture veratrum viride is about one-seventh weaker than formerly.

Wine of ipecac is between one-fith and one-fourth stronger than formerly.—*From a recent address by Dr. H. H. Rusby.*

---

### TO DETECT CASTOR OIL IN OLIVE OIL.

S. Leonardi (*Pharm. Ztg.* 1893; p. 705) recommends the following very simple method: ten c. c. of the suspended oil are vigorously shaken with $\frac{5}{10}$ c. c. of alcohol (94-100 per cent.) and then set aside until the two layers have become completely separated. If the olive oil is pure its volume will increase and that of the alcohol decrease; but, if it contains castor oil the contrary will occur, that is, the volume of the oil will decrease and that of the alcohol increase. For a quantitative estimation, alcohol is employed which has previously been saturated with olive oil and filtered; the increase in volume of the alcohol will then correspond exactly to the quantity of castor oil contained in the olive oil. The presence of castor oil is furthered by mixing the suspected oil with concentrated alcoholic potassa-solution and heating it, then the peculiar odor of caprglic alcohol will appear.

---

### LIQUEFIED CARBOLIC ACID.

At a recent meeting of the British Pharmaceutical Society, Peter Boa called attention to the difficulty of keeping liquefied carbolic acid (one part of water to nine of acid) fluid in cold weather. Whenever the temperature of the pharmacy fell below 50° F., he said crystallization may be expected. For some time he had been replacing a portion of the water by alcohol with good results. One hundred parts of carbolic acid, seven and a half parts water and two and a half parts alcohol give a liquefied carbolic acid that remains fluid in the shop bottle till the temperature falls to about 40° F. A larger proportion of spirit does not appear to serve the purpose better. When this liquefied acid, containing two and a half parts of spirit in place of two and a half parts of water, crystallizes, the resulting mass is less hard and much more easily reliquefied than the mass which results on crystallization when water only is used to liquefy ; it is as snow compared with ice.

---

### TEST FOR SALOL.

It is reported (*Journ. de Pharm. d' Anvers*) that if to a small quantity of salol (in a porcelain capsule) there be added a few drops of nitro-sulphuric acid, the mixture will first turn yellow, then brown, and finally green. If water be added to this mixture, it turns reddish on stirring ; and if ammonia be admixed the color will change to greenish. On treating resorcin in the same manner, there first appears a beautiful dark blue coloration, turning red on the addition of water and changing again to blue on adding ammonia.

---

### TEST FOR MINERAL ACIDS IN VINEGAR.

Grizzi (*Chem. Zeit.*) recommends placing 1 c. c. of the suspected vinegar in a shallow china saucer, and adding one drop of a solution of 1 gram of fuchsin in 4 c. c. of 90 per cent. alcohol. If the vinegar be pure, the purple red color of the fuchsin will deepen after the fluids have mingled, but will be otherwise unaltered, while it will change to dirty yellow if even 1 per cent. of mineral acid be present. The fuchsin color can be restored by neutralization with an alkali.

